# Impact of Wild Edible Fruits of *Arbutus unedo* and *Crataegus monogyna* on Gut Motility, Contraction, Secretion, and Glucose Regulation

**DOI:** 10.1111/nmo.70189

**Published:** 2025-10-27

**Authors:** Soumaya Wahabi, Kais Rtibi, Chaima Abidi, Mourad Jridi, Bernard Gressier, Hichem Sebai, Bruno Eto

**Affiliations:** ^1^ University of Jendouba, Higher Institute of Biotechnology of Beja LR: Functional Physiology and Valorization of Bio‐Resources Beja Tunisia; ^2^ Laboratory of Pharmacology, Pharmacokinetics and Clinical Pharmacy, UFR3S, Dept of Pharmacy University of Lille Lille France; ^3^ Laboratories TBC, Laboratory of Clinical Pharmacy, UFR3S Dept of Pharmacy University of Lille Lille France

**Keywords:** *Arbutus unedo*, *Crataegus monogyna*, GI‐motility/dysmotility, hyperglycemia management, rats, spontaneous jejunal smooth muscle contractility

## Abstract

**Background:**

Arbutus and hawthorn fruits are commonly utilized in traditional medicine to address various gastrointestinal (GI) ailments. Our primary aim was to individually assess the effects of aqueous extracts from *Arbutus unedo* (AUAE) and *Crataegus monogyna* (CMAE) fruits on GI motility, spontaneous jejunal smooth muscle contractility, and hyperglycemia management.

**Methods:**

Wistar rats were administered loperamide (LOP, 3 mg/kg, b.w.) along with AUAE or CMAE (at doses of 75, 150 and 300 mg/kg, b.w.) or yohimbine (YOH, 2 mg/kg, b.w.). GI transit was evaluated using the charcoal meal test. The impact of both extracts on jejunal secretion and contraction was assessed using the Ussing chamber technique and the isometric transducer. The bioactive constituents of AUAE and CMAE were analyzed via liquid chromatography‐high resolution electrospray ionization mass spectrometry (LC‐HRESIMS).

**Results:**

AUAE and CMAE comprise bioactive compounds, including phenolic acids, flavonoids, and flavonols, capable of eliciting various intended physiological effects. Both extracts demonstrated a significant and dose‐dependent increase in GI transit (77.33%–89.83% and 80.31%–85.54%, respectively) compared to the delayed peristalsis induced by LOP (42.77%) and the accelerated effect of YOH (90.09%). Both extracts induced an increase in the amplitude of spontaneous jejunal contraction with an EC_50_ of 90.47 and 22.98 μg/mL, respectively. Conversely, the two extracts did not impact the electrogenic transport of intestinal fluid when compared to the action produced by forskolin (FSK, 10 μM). Additionally, both extracts significantly reduced glucose levels in hyperglycemic rats compared to control values.

**Conclusion:**

These findings hold promise for the development of novel preventive and pharmacological treatment strategies for GI disorders and diabetes management.

## Graphical abstract

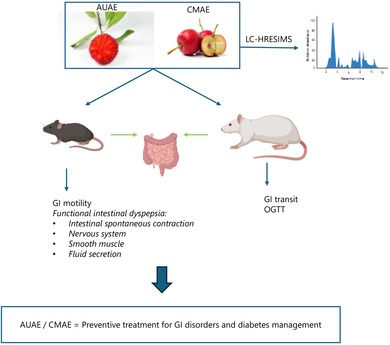

**Summary**

- The aqueous extracts of *Arbutus unedo* and *Crataegus monogyna* fruits significantly enhanced gastrointestinal motility and increased spontaneous jejunal smooth muscle contraction in rats.
- Both extracts reduced blood glucose levels without affecting intestinal secretion.
- LC‐HRESIMS analysis identified phenolic acids and flavonoids as major bioactive compounds.
- These findings suggest that these wild edible fruits may offer natural therapeutic potential for gastrointestinal dysmotility and hyperglycemia management.

**Abbreviations**
AUAE:
*Arbutus unedo* aqueous extract
CA: Cinnamic acid
Carbchol: Carbamylcholine
CMAE: *Cratagusmonogyna* aqueous extract
EC_50_
: half maximal effective concentration
FSK: Forskolin
GIT: Gastrointestinal transit
IC_50_
: half‐maximal inhibitory concentration
Isc: Short circuit current
LOP: Loperamide
QR: Quercetin
SCFAs: Short‐chain fatty acids
STC: Slow transit constipation
YOH: Yohimbine

## 1 Introduction

Dyspepsia is a chronic disorder marked by symptoms in the upper gastrointestinal tract [1]. It is also a clinical term used to define the sensation of difficult digestion. Some of the most common symptoms are postprandial fullness, discomfort, early satiety, bloating, belching, nausea, vomiting, or pain. Dyspepsia can be classified as ‘organic’ when an underlying organic disease is likely to be causing the symptoms and as functional dyspepsia when no organic abnormality is identified [2].

Small intestinal glucose absorption management is an important target for the control of blood glucose levels [3]. For this, metformin, a biguanide anti‐hyperglycemic agent, accumulates in the intestinal mucosa and increases glucose turnover, providing its antihyperglycaemic effect [4]. In isolated rat jejunal, it reduced the glucose‐induced short‐circuit current, thus inhibiting the activity of sodium‐glucose transporter 1 (SGLT1) accompanied by recruitment increase of the glucose transporter 2 (GLUT2) to the apical membrane of the rat jejunum [5]. Since metformin boosts intestinal glucose uptake and lactate production [6, 7], this phenomenon can cause intolerance to the treatment [7] and contribute to the development of lactic acidosis [3].

In Tunisia, herbal medicines play an important role in traditional medicine, which is largely used in various areas of health [8]. These plants are considered to be an important source of new chemical substances with potential therapeutic [9, 10].

The strawberry tree *(Arbutus unedo L.)* belongs to the family of *Ericaceae. It* grows well on the Iberian Peninsula, in the Mediterranean basin, and in other regions with warm summers and mild, rainy winters [11]. The fruit is spherical, about 2–3 cm in diameter, red, and tasty only when fully ripe. They are edible but usually processed before consumption [11]. *A. unedo* fruits are a rich source of health benefits, notably vitamin C and dietary fiber, and are also considered a source of bioactive compounds for nutritional supplements or functional foods [12]. They contain phenolic acids and flavonols [13], as well as a large quantity of vitamins [14], fatty acids [15, 16], sugar [17, 18], and minerals [19].

*Crataegus monogyna* belongs to the family of *Rosaceae*. It is among the most highly recommended species in traditional medicine, and the berries are generally eaten by shepherds, hunters, and children, as they are considered ‘healthy’ and nutritious for both humans and animals [20]. *C. monogyna* has traditionally been used to treat diarrhea, gall bladder disease, and insomnia [21], as well as respiratory problems such as coughs, flu, bronchitis, and asthma [22]. Hawthorn has been used in Europe to treat heart problems due to its antispasmodic, cardiotonic, hypotensive, and anti‐atherosclerotic effects [23]. In addition, in traditional Chinese medicine, hawthorn has been used to treat heart problems [24].

To confirm that both fruits are used as medicine traditionally to prevent/treat GI disorders, in this study, we examined their modulatory actions on GI motility, spontaneous ex vivo jejunal contraction/secretion, and glucose tolerance.

## 2 Materials and Methods

### 2.1 Chemicals

All reagents were purchased and used for research only. Carbacholine hydrochloride, D‐glucose, D‐mannitol, metformin, phloridzin, bumetanide, ouabain, and forskolin were purchased from Sigma (Sigma–Aldrich).

### 2.2 AUAE and CMAE Preparation

*A. unedo and C. monogyna* fruits were collected from the area of Beja, northwest of Tunisia, during October and November 2023 and identified by the botanic coordinator Chokri Hafsi, Institute of Biotechnology of Beja, University of Jendouba. Thereafter, the fruit material (all the fruit for *A. unedo* plant and the pericarp for the *C. monogyna* plant) was dried at 40°C for a duration of 5 days with air circulation and later rigorously crushed in a traditional metallic mortar and then with an electric blender. Aqueous extracts of arbutus and hawthorn fruits (AUAE and CMAE) were prepared by adding the powder to boiled bi‐distilled water (70°C), and the mixture was stirred for 1 h. The aqueous extracts were filtered using a Buchner funnel and Whatman No. 1 filter paper. The filtrates were quickly frozen at 40°C and dried for 48 h using a freeze dryer (Refrigerated Vapor Trap RVT450).

### 2.3 *AUAE and CMAE Phenolic Compounds Identification by Liquid Chromatography‐*High Resolution Electrospray Ionization Mass Spectrometry (LC‐HRESIMS) Analysis

100 mg of each extract were dissolved in 100 mL of 10% methanol, filtered, and then 1 mL was transferred into LC–MS vials. An opposite‐phase column (Pursuit XRs ULTRA 2.8, C18, 100 × 2 mm, Agilent Technologies, UK) was used to carry out HPLC investigations. 20 mL of the prepared samples was injected at a column temperature set at 30° *C. Mobile* phases consisted of 0.1% formic acid in water (A) and 0.1% formic acid in methanol (B). A gradient program was used for isolation at a flow rate of 1 mL min^−1^. Mobile phases consisted of an initial composition of 100% solvent A, with a gradient of 100% solvent B over 20 min, held at 100% solvent B for 5 min, and 100% solvent A for 25 min. The drying gas flow rate was 1 mL min−1 at 320°C. MS was operated in the positive ion mode in a mass range of 100–2000 m/z. High‐resolution mass spectral data were obtained on a Thermo Instruments ESI‐MS system (LTQ XL/LTQ Orbitrap Discovery, UK) connected to a Thermo Instruments HPLC system (Accela PDA Detector, Accela PDA Autosampler, and Accela Pump) [25].

### 2.4 In Vitro Antimitotic Activity

Watercress seeds, reference BA 902, are germinated in petri dishes on a layer of filter paper at room temperature in increasing doses of the following extracts and hawthorn fruit extract (0.1 μg/mL to 50 mg/mL) at a rate of 12 seeds per box. After 72 h, the length of the rootlets was measured using a graduated ruler.

### 2.5 Used Animals for Experiments

Adult male rats of the Wistar strain (ten weeks old, weighing 180–220 g) were purchased from the Society of Pharmaceutical Industries of Tunisia (SIPHAT, Ben‐Arous, Tunisia). The animals were housed six per cage with ad libitum access to water and standard food (Badr‐Utique‐TN). They are maintained under standard conditions of temperature 22°C ± 2°C, relative humidity of 50%, and 12 light/dark cycles in the animal house of the Higher Institute of Biotechnology of Beja and used for intestinal transit studies.

Male mice of the C57BL/6JRj strain (7 weeks old, weighing 20–25 g) from Janviers SASA (Route des Chenes, Le Genest‐st‐Isle, St Berthevin, France) were grouped in polycarbonate cages and acclimatized for 1 week under the following conditions (22°C–26°C, ventilation, and 12/12 light/dark cycle) with free access to water and food in the animal house of the Faculty of Pharmacy, University of Lille, France, and used for intestinal contraction studies. Circadian rhythm can influence important functions in the body; for this, all experiments were performed at the same time (9 h) every day. All animals were handled in accordance with internationally accepted guidelines [26].

### 2.6 GI‐Transit Measurement

GI‐motility was evaluated using the charcoal meal method [27]. The rats were fasted for 16 h and divided into 9 groups of 6 animals each: Group 1 served as a negative control and received 1 mL of physiological solution (NaCl, 0.9%); Groups 2 and 3 received YOH (2 mg/kg, b.w.) and LOP (3 mg/kg, b.w.), respectively; Groups 4, 5, and 6 were treated with different doses of the AUAE (75, 150 and 300 mg/kg, b.w.); and Groups 7, 8, and 9 were treated with different doses of the CMAE (75, 150 and 300 mg/kg, b.w.). A standard charcoal meal (10% charcoal in 5% gum arabic) was administered orally using an intra‐gastric tube 2 h after treatment. 30 min later, the animals were sacrificed and the distance traveled by the charcoal meal from the pylorus was measured. GIT was expressed as a percentage and calculated according to the following rule:

$$\begin{array}{cc} \mathrm{GIT}\;\left(\%\right)\; & =\text{Distance traveled}\;\mathrm{by}\;\text{charcoal meal}\\ & /\text{Length of small intestine}\;\left(\mathrm{cm}\right)\times \;100 \end{array}$$

### 2.7 Study of Jejunal Contraction and Relaxation

Overnight fasted male mice are subject to vertebral dislocation. A segment of 5 cm of the jejunum was excised and washed in saline solution under ice. Forceps were used to strip off precisely the jejunum mesenteric border. The jejunum sections (2 cm) were detached using flushing with a solution of Tyrode, whose composition is as follows: NaCl (136.9 mM), KCl (2.7 mM), CaCl_2_ (1.8 mM), NaHCO_3_ (11.09 mM), MgCl_2_ (1.05 mM), NaH_2_PO_4_ (0.42 mM), and glucose (5.5 mM) at pH 7.4. Each tissue was put in a 3 mL organ bath enclosing Tyrode's solution maintained at 37°C ± 0.5°C and providing 95% O_2_ and 5% CO_2_. A first tension of 0.5 g was used, and the spontaneous muscular contractility was registered isometrically at the same time utilizing JFD‐2 Transducer (Laboratories TBC, France). Drugs, AUAE and CMAE, were joined immediately to the organ chamber in volumes not more than 5% of the total bath volume. At the last of the 45‐min equilibration duration, the actions of various doses of AUAE, CMAE, and/or the drugs were evaluated cumulatively with a contact period of 2 min for each concentration. The effect on contraction and relaxation of the extract at a concentration of 500 μg/mL against 10^−6^ M carbamylcholine (CarbCh), 25 mM KCl, and 10 mM CaCl_2_ was assessed.

### 2.8 Ex Vivo Intestinal Tissue Preparation and In Vitro Short‐Circuit Measurement

The spontaneous transmural electrical potential difference (PD), reflecting the asymmetry of electrical charges between the luminal and serosal intestinal mucosa, was measured using agar bridges containing 3 M KCl solution in 4% agar (w/v). These bridges were positioned on both sides of the tissue and connected to calomel half‐cells, which were linked to a high‐impedance voltmeter. The PD was short‐circuited and maintained at 0 mV throughout the experiment by a short‐circuit current (I_sc_), facilitated via two stainless steel 316 L working electrodes directly placed in each reservoir. This setup was connected to a voltage clamp system (JFD‐1 V, Laboratoires TBC, France).

The delivered Isc, corrected for fluid resistance, was continuously recorded using Biodaqsoft software (Laboratoires TBC, France). The Isc (in μA/cm^2^) represents the sum of net ion fluxes transported across the epithelium, predominantly Na^+^, Cl^−^, and HCO_3_‐, in the absence of an electrochemical gradient [28].

Stock solutions of plant extracts were prepared extemporaneously to achieve final concentrations ranging from 1 to 1000 μg/mL. These solutions were added to either the serosal or mucosal chamber (prewarmed at 37°C) in a volume of 20–40 μL, 3 min before the addition of luminal glucose (on the serosal side of the tissue, glucose was replaced with mannitol).

Results were expressed as the difference (ΔIsc) between the peak Isc after glucose challenge (measured after 20 min) and the Isc measured just 5 min after the addition of AUAE and CMAE extracts, followed by the addition of 0.5 mM of phloridzin (PHZ) in single concentration experiments or after an AUAE and CMAE concentration series in a cumulative concentration experiment [19]. The percent of inhibition referred to individual values compared to the maximal inhibition induced by PHZ (100%).

### 2.9 Oral Glucose Tolerance Test (OGTT)

Rats were fasted for 16 h before being subjected to an OGTT by intragastric gavage with a glucose solution to achieve a glucose load of 2 g kg^−1^. OGTT was carried out 3 h after the initial extract administration. Blood samples were collected from the tail vein at 0, 15, 30, 60, 90, 120, 150 and 180 min, and blood glucose was determined using a glucometer (Accu‐Chek, Roche, QC, Canada) [29].

### 2.10 Statistical Analysis

All data are presented as the mean ± S.E.M of the indicated number of experiments. Results were analyzed by one‐way or two‐way analysis of variance (ANOVA) with a Tukey–Kramer multiple comparisons posttest performed using GraphPad Prism version 5.01 for Windows (Graphpad software Inc., San Diego, CA). GraphPad Prism computes the area under the curve using the trapezoid rule. Statistical significance was set at *p* < 0.05.

## 3 Results

### 3.1 Absorbance Spectrum of AUAE and CMAE

**FIGURE 1:**
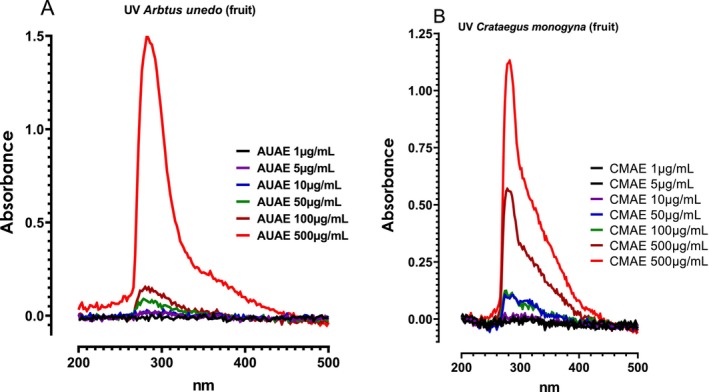
Absorbance spectrum of aqueous extracts of *Arbutus unedo* fruit (A) and Hawthorn (B) at different concentrations (1, 5, 10, 100, 500 and 1000 μg/mL).

The UV–Visible absorption spectrum of the extracts at different concentrations (1, 5, 10, 100, 500 and 1000 μg/mL) is shown in Figure 1. UV spectral examination shows an absorption band at 280 nm. The different concentrations of two extracts absorb at the same wavelength, which is around 280 nm.

### 3.2 Identification and Quantification of AUAE and CMAE Phenolic Compounds With LC‐HRESIMS Assay

The LC‐HRESIMS technique enabled us to identify 11 phenolic compounds in AUAE and 6 phenolic compounds in CMAE (Table 1). In fact, there are two main classes of polyphenols, such as phenolic acids and flavonoids (quercetin glycosides, flavanols, flavonols, and flavanones). Among the phenolic acids present in AUAE, we found chlorogenic acid, syringic acid, sinapic acid, ferulic acid, and cinnamic acid. However, the remaining compounds belong to different classes, such as flavonoids, including catechin, quercetin glycosides (rutin), flavonols (myricetin and quercetin), and flavanones (naringenin). However, six compounds were detected in the CMAE. Phenolic acids were represented by three compounds (syringic acid, sinapic acid, and ferulic acid). Flavonoids were represented by catechin, myricetin, and quercetin.

**TABLE 1:** Identification of phenolic compounds in AUAE and CMAE using the LC–MS technique.

NO^*a*^	Compounds^*b*^	Molecular formula	Molecular mass	[M‐H] −m/z	Retention time (min)	AUAE	CMAE
1	Resorcinol	C_6_H_6_O_2_	110	109	11.554	4.00	—
2	Chlorogenic Acid	C_16_H_18_O_9_	354	353	11.996	2.30	—
3	Catechin	C_15_H_14_O_6_	290	289	12.561	3.28	5.26
4	Syringic Acid	C_9_H_10_O_5_	198	197	14.609	2.97	20.80
5	Rutin	C_27_H_30_O_16_	610	609	16.726	76.50	—
6	Sinapic Acid	C_11_H_12_O_5_	170	169	18.840	2.21	3.90
7	Ferulic Acid	C_10_H_10_O_4_	194	193	19.206	3.04	18.39
8	Myricetin	C_15_H_10_O_8_	318	317	22.546	2.4	40.14
9	Quercetin	C_15_H_10_O_7_	302	301	26.161	1.11	11.63
10	Cinnamic Acid	C_9_H_8_O_2_	148	147	27.546	1.28	—
11	Naringenin	C_15_H_12_O_5_	272	271	28.834	1.01	—

### 3.3 In Vitro Antimitotic Activity

**FIGURE 2:**
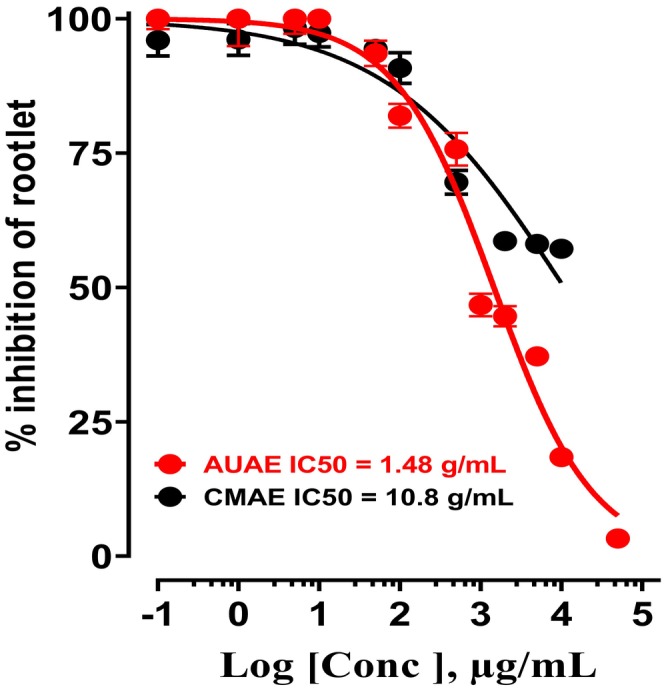
Inhibition of *Lepidium sativum* germination (rootlet growth) in the presence of different concentrations of AUAE and CMAE.

According to the results shown in Figure 2, we found that the aqueous extract of arbutus fruit can inhibit the growth of watercress rootlets at the highest concentrations, with an IC_50_ value of 1174 μg/mL, whereas the aqueous extract of hawthorn fruit has a partial effect.

### 3.4 GI Transit

**FIGURE 3:**
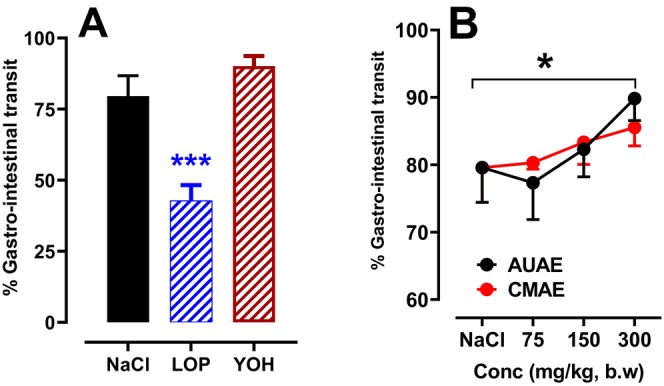
Effect of AUAE and CMAE on GIT. Data are expressed as means ± SEM (*n* = 6). *: *p* < 0.05, ***: *p < 0.001* in comparison with the no treated animals (ANOVA test). As a negative control, an animal received 1 mL of physiological solution (NaCl, 0.9%); Groups 2 and 3 received YOH (2 mg/kg, b.w.) and LOP (3 mg/kg, b.w.), respectively. Groups 4, 5, and 6 were treated with different doses of the AUAE (75, 150 and 300 mg/kg, b.w.), and Groups 7, 8, and 9 were treated with different doses of the CMAE (75, 150 and 300 mg/kg, b.w.).

The effect of plant extracts on gastrointestinal movement is demonstrated in Figure 3. A significant decrease in gastrointestinal movement was observed in LOP‐group rats compared to the negative control, with a reduction of 46.24%. Therefore, LOP delayed gastrointestinal transit. In contrast, AUAE and CMAE showed a significant increase in gastrointestinal transit speed in a dose‐dependent manner. Comparing the effect of the two extracts, a non‐significant difference was also observed in GIT.

### 3.5 Effects of AUAE and CMAE on Intestinal Smooth Muscle Contraction

#### 3.5.1 Effect of AUAE on Intestinal Spontaneous Contraction

**FIGURE 4:**
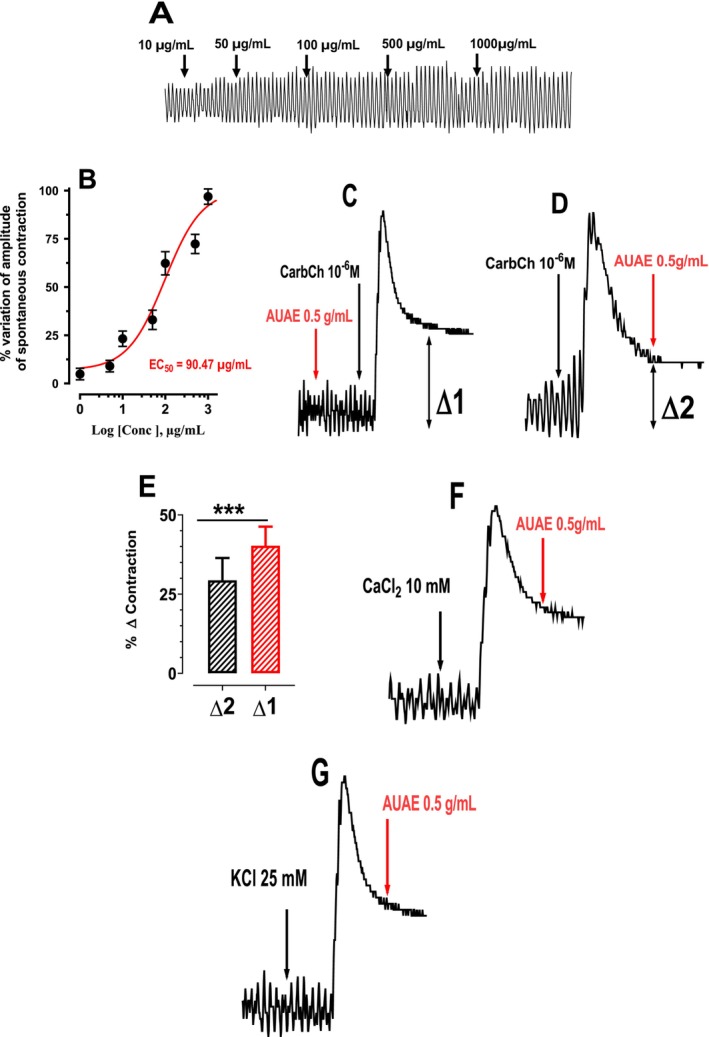
(A) Typical recording of the effect of aqueous extract of *Arbutus unedo* fruit on spontaneous contraction of the mouse jejunum. (B) Concentration‐response effect of AUAE (1–1000 μg/mL). This figure shows that the concentration of AUAE which induces 50% of the maximum concentration (EC_50_) is 90.47 μg/mL. The concentration–response curve was obtained using non‐linear regression using Hill's equation by an iterative least‐squares method. Effect of aqueous extract of *Arbutus unedo* fruit (AUAE 500 μg/mL) on jejunal contraction induced by Carbamylcholine (CarbCh 10^−6^ M) before (C) and after (D) stimulation, after stimulation with CaCl_2_ (10 mM) (F) and KCl (25 mM) (G). (E) Percentage variation in contraction induced by CarbCh before and after the administration of AUAE (***: *p < 0.001*).

The results obtained in Figure 4A,B showed that the AUAE does not induce intestinal relaxation or contraction. However, AUAE induces an increase in the amplitude of spontaneous contraction of the intestine as a function of various doses (1, 10, 50, 100, 500 and 1000 μg/mL) (Figure 4A) with EC_50_ of 90.47 μg/mL (Figure 4B).

#### 3.5.2 Effect of AUAE on Carbochol‐Induced Intestinal Contraction

Figure 4C–E shows that AUAE does not inhibit the contraction induced by CarbCh, which activates acetylcholine receptors as a cholinergic agonist (Figure 4C,D). Moreover, Figure 4E indicates that when arbutus is administered prior to CarbCh, it enhances the contraction induced by CarbCh. A similar effect was observed when contractions were induced by histamine (10^−6^ M) and serotonin (5‐HT, 10^−5^ M), though these results are not shown. These findings suggest that AUAE does not act as an inhibitor of serotonin, histamine, and acetylcholine receptors but can modify their effect. Indeed, the absence of an effect of AUAE on contraction induced by CarbCh, histamine, or 5‐HT does not allow for the conclusion that it does not act directly on smooth muscle. It is possible that AUAE does not interfere with these specific contraction pathways, or that other underlying mechanisms, not tested under these conditions, could be involved. Therefore, this observation does not exclude the potential for a direct effect of AUAE on smooth muscle, which may be revealed under different experimental conditions.

In addition, we noted that the amplitude of Carbachol‐induced contraction of the intestine was slightly reduced when AUAE was introduced into the preparation after the Carbachol‐induced contraction. This suggests a partial modulation of AUAE on the carbachol‐induced contraction, although it does not imply complete inhibition.

#### 3.5.3 Effect of AUAE on Contraction Induced by CaCl_2_ and KCl

The results presented in Figure 4F,G show that aqueous AUAE did not inhibit contractions induced by CaCl_2_ (10 mM) (Figure 4F) or KCl (25 mM) (Figure 4G).

#### 3.5.4 Effect of CMAE on Spontaneous Contraction of the Intestine

**FIGURE 5:**
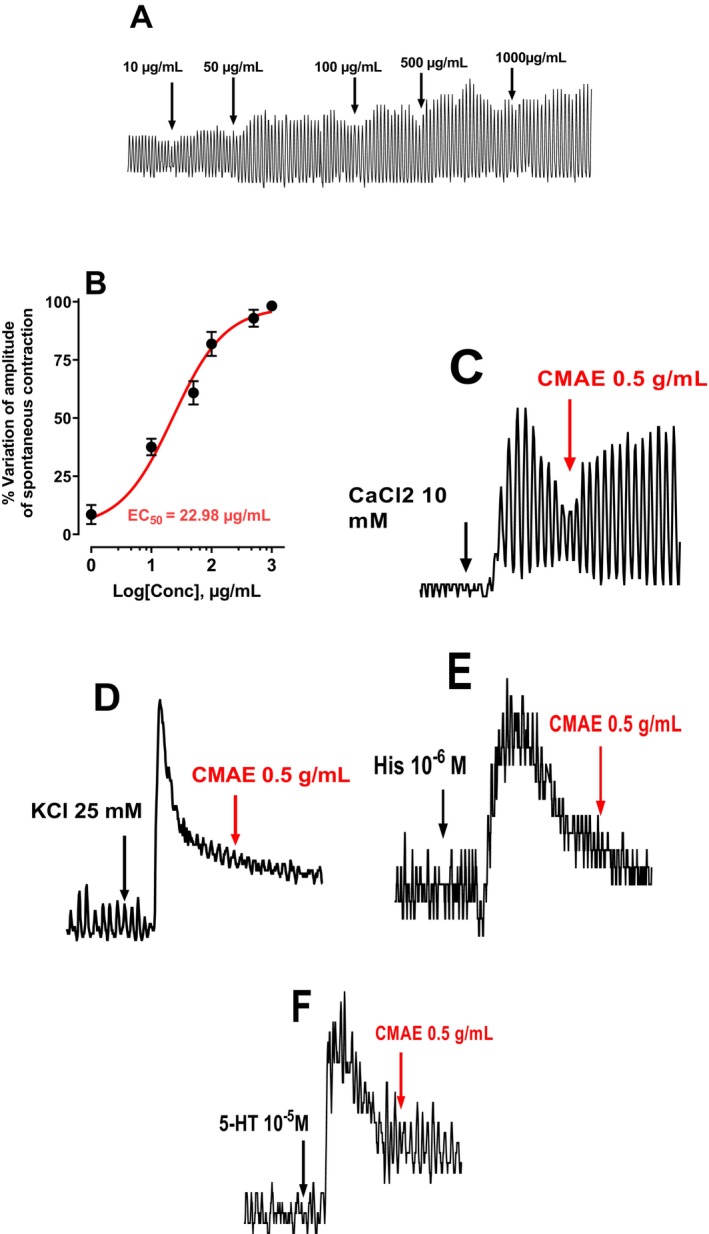
(A) Typical recording of the effect of aqueous extract of *Crataegus monogyna* fruit on spontaneous contraction of the mouse jejunum. (B) Concentration‐response effect of CMAE (1–1000 μg/mL). This figure shows that the concentration of CMAE which induces 50% of the maximum concentration (EC_50_) is 22.98 μg/mL. The concentration–response curve was obtained using non‐linear regression using Hill's equation by an iterative least‐squares method. Effect of aqueous extract of *Crataegus monogyna* fruit (500 μg/mL) after stimulation with CaCl_2_ (10 mM) (C), KCl (25 mM) (D), histamine (10^−6^ M) (E), and serotonin (10^−5^ M) (F).

The results obtained in Figure 5A showed that the CMAE does not induce intestinal relaxation or contraction. However, CMAE induces an increase in the amplitude of spontaneous contraction of the intestine as a function of various doses (1, 10, 50, 100, 500 and 1000 μg/mL) with EC_50_ of 22.98 μg/mL (Figure 5B).

#### 3.5.5 Effect of CMAE on Contraction Induced by CaCl_2_ , KCL and Serotonin and Histamine

Figure 5C,D shows that the aqueous extract of Hawthorn fruit did not reduce the contraction induced by either CaCl_2_ (25 mM) (Figure 5C) or KCl (10 mM) (Figure 5D), Figure 5E,F shows that the aqueous extract of hawthorn fruit does not reduce the contraction induced by histamine (10^−6^ M) (Figure 5E) and serotonin (5‐HT 10^−5^ M) (Figure 5F). CaCl_2_, KCl, histamine, and serotonin have both neuronal and direct smooth muscle actions. Although they may give us some indication of the neuronal or smooth muscle effects of CMAEs or AUAEs, further studies are needed to conclusively determine their precise actions. In this experiment, we were unable to obtain this certainty of the action of CMAE as AUAE on the neuronal or muscular system.

### 3.6 Effects of AUAE and CMAE on Intestinal Secretion (Ussing Chamber)

**FIGURE 6:**
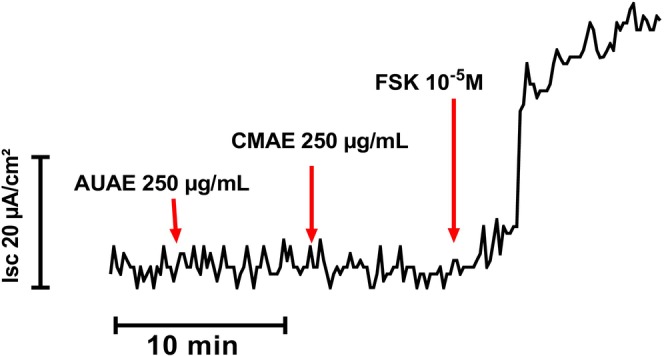
Typical recording of the short‐circuit current after the addition of different concentrations of AUAE, CMAE, and Forskoline (FSK).

The typical recording of the short‐circuit current after the addition of different concentrations of AUAE and CMAE shows that the plant extracts have no effect on the short‐circuit current (*Isc*). Similarly, these two extracts had no effect on the increase in short‐circuit current induced by Forskolin (Figure 6). Therefore, AUAE and CMAE do not influence the intestinal secretion of water and electrolytes induced by FSK, which rules out their effects as a secretagogue and anti‐secretagogue.

### 3.7 Oral Glucose Tolerance Test

**FIGURE 7:**
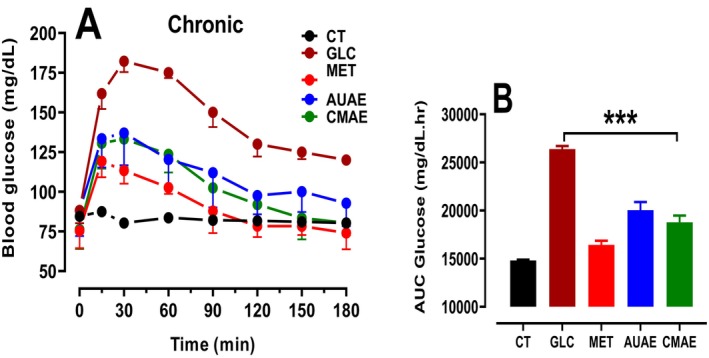
OGTT after chronic AUAE and CMAE oral treatment in rats. Rats were gavaged intragastrically with aqueous plant extracts (300 mg/kg bodyweight) and OGTT (glucose, 2 g/kg bodyweight). (A) Blood glucose levels over time. (B) Area under the curve (AUC) for blood glucose levels in the different experimental groups. Significantly different from control (glucose): ***p < 0.001.

Hyperglycaemia was induced in healthy rats by intragastric gavage of a glucose solution (2 g/kg) 30 min after administration of the aqueous plant extract (AUAE or CMAE) at a dose of 300 mg/kg body weight. The variation of blood glucose levels was measured at increasing time intervals (0, 15, 30, 60, 90, 120, 150 and 180). A peak in hyperglycaemia was observed immediately after the animals were gavaged with the glucose solution. 3 h after the animals were treated with the extracts, we observed a correction in hyperglycaemia. Our results showed that an acute oral administration of AUAE and CMAE significantly reduced the glucose concentration in rats as compared to control values (glucose) (Figure 7). This effect was also observed in the group of animals after the administration of metformin.

## 4 Discussion

Currently, plants occupy an important place in therapeutic approaches and new drugs used against diseases. In the present study, our objective is to assess, in a separate way, the in vitro antimitotic capacity of AUAE and CMAE and their modulatory actions on GI‐motility, ex vivo spontaneous jejunal smooth muscle contractility, and in vivo provoked hyperglycemia. Chromatographic analysis of plant extracts using HPLC identified 11 phenolic compounds in AUAE (chlorogenic acid, rutin, resorcinol, catechin, ferulic acid, syringic acid, myricetin, sinapic acid, cinnamic acid, quercetin, and naringenin). Similarly, 6 phenolic compounds were identified in the CMAE (myricetin, syringic acid, ferulic acid, quercetin, catechin, and sinapic acid). Chlorogenic acid was a very common element in the AUAE, and numerous studies on the chemical composition of strawberry fruits have amply confirmed their richness in this molecule [30, 31]. It is an important and biologically active dietary polyphenol with several therapeutic roles such as antioxidant, antibacterial, hepatoprotective, cardioprotective, anti‐inflammatory, antipyretic, neuroprotective, anti‐obesity, antiviral, antimicrobial, anti‐hypertensive, free radical scavenger, and central nervous system stimulatory activity [32]. In addition, chlorogenic acid has been shown to modulate lipid and glucose metabolism in metabolic disorders [32]. Previous studies have shown that CMAE contains high levels of many valuable secondary metabolites, including flavonoids, vitamin C, glycosides, anthocyanins, saponins, and tannins [33, 34]. Myricetin is a flavonoid compound widely found in the CMAE. To date, myricetin has been shown to have multiple biological functions and is mainly used for its anti‐inflammatory [35], antitumor [36], antibacterial [37] and antiviral [38] effects. It also exerts cardiovascular protection [39], protects against neurological damage [40] and protects the liver from potential damage [41]. Antimitotic activity of AUAE and CMAE was studied against watercress seeds. Our results showed for the first time that AUAE can inhibit the growth of watercress rootlets at the highest concentrations, with an IC_50_ value of 1174 μg/mL. The antimitotic activity observed with AUAE could result from the involvement of certain phytochemical compounds, notably phenolic compounds and flavonoids. Phenolic compounds have an inhibitory effect on the activation metabolism of potential carcinogenic substances. Their antimitotic action is due to their ability to prevent cell division during telophase [42]. In this context, a concentration‐dependent antiproliferative action of myricetin has been reported in human papillary thyroid cancer cells (SNU‐790), revealing cytotoxicity, causing DNA condensation, upregulating the Bax: Bcl‐2 ratio, inducing caspase cascades, modifying the mitochondrial membrane potential, and tempting the apoptosis‐inducing factors release [43].

Functional intestinal dyspepsia may be recognized by a set of chronic digestive symptoms revealing malfunction of the upper digestive tract, provoked or accentuated in a variable way by food, without any organic lesion being noted. Depending on the case, these symptoms are accompanied by poor relaxation of the stomach which impairs its ability to act as a reservoir when food arrives, a slowing of gastric emptying (mainly during the solid phase of the meal), and/or gastric or even duodenal mechanical or chemical hypersensitivity [44]. In the present work, AUAE and CMAE are significantly able to increase GIT in a dose‐dependent manner, particularly with the highest dose (300 mg/kg, b.w.). A non‐significant increase in GIT was observed in the AUAE group compared with the CMAE group. The laxative effect of the AUAE and CMAE may be due to the richness of the fruit in total dietary fiber [45]. The importance of fiber for the normal function of the digestive system has been long appreciated. Considering the recommendation of fiber intake, which is 21–38 g/day for adults [46], the intake of 100 g of strawberry‐tree fruits could provide 30%–40% of the daily recommendation of fiber. According to European Regulations, *A. unedo* fruits could be considered under the approved mention “high in fiber” [47]. On the other hand, the richness of the fruit in celluloses, lignins, and pectins may be the cause of the laxative effect of the CMAE [48]. Fiber densifies stools, making them bulkier and allowing them to retain water, which stimulates natural peristalsis and, consequently, stool movement. For these reasons, fibers accelerate colonic motility [49].

On the other hand, the ferulic acid action on peristalsis and gastric emptying further supports the gastrokinetic effect of this compound. Indeed, Badary et al. showed that indomethacin could reduce the facilitatory effect of ferulic acid, which further indicates the partial prostaglandins involvement in the mechanism of action of ferulic acid on gastrointestinal motility [50]. Very recent research demonstrated that Cinnamic acid (CA) was an effective agent in treating the slow transit constipation (STC). These results indicated that CA ameliorated the infiltration of neutrophils and lymphocytes, increasing the number of goblet cells and the colon mucosa secretory function. CA significantly improved the diversity and abundance of the beneficial microbiome. Furthermore, the changed abundance of Firmicutes, Akkermansia, Lachnoclostridium, Monoglobus, UCG.005, Paenalcaligenes, Psychrobacter, and Acinetobacter were implicated in the generation of short‐chain fatty acids (SCFAs). These data showed that CA could ameliorate the composition and abundance of the intestinal microbiome to regulate the production of SCFAs in STC [51]. CA can indeed induce contraction and relaxation of rat ileum smooth muscles in a dose‐dependent manner, and its effects may involve various pathways, including cholinergic, adrenergic, non‐adrenergic, non‐cholinergic, and TRP channels [52].

In this context, other data showed that quercetin (QR) present in *Amomum villosum Lour* has the most prominent relieving action on STC. It can produce this effect by promoting digestion and absorption of GI tract contents, promoting gastrointestinal tract water and electrolyte balance, and promoting gastrointestinal tract peristalsis. QR elevated GI hormone levels and balanced the relative abundance of beneficial and harmful bacteria and the gut microenvironment to promote digestion and absorption of contents in the gut. QR facilitated elimination of the contents by increasing the water of the contents via modulating GI hormones, which also facilitated the inward flow of Ca^2+^ through the CAM‐MLCK pathway, thus enhancing GI motility levels. Furthermore, QR enhances the abundance of gut microbiota, restores the neuro‐neurotransmission of the enteric nervous system, and promotes intestinal motility [53].

Yohimbine, an alpha‐2 adrenergic antagonist, significantly inhibited loperamide‐induced constipation. However, in this study, it has almost the same effect as AUAE. Our results also clearly show that AUAE and CMAE increase the amplitude of spontaneous contraction of the mouse jejunum intestine. It is well known that most pharmacological tools that inhibit the enteric nervous system reduce the amplitude of spontaneous contraction of the intestine or suppress it. Apparently, everything depends on the tonic activities of the excitatory or inhibitory neurons. In the animal species with tonic inhibitory activity, tetrodotoxin enhances intestinal contraction by blocking the inhibitory signals [54].

We have shown that when the intestine is stripped (removed of external longitudinal muscle), the amplitude of spontaneous contraction of the intestine is greatly reduced or even suppressed. Only the mesenteric edge was removed, while the remaining muscle and nerve layers were left intact, allowing for their subsequent stimulation with pharmacological agents.

The fact that AUAE increases the amplitude of spontaneous contraction of the intestine might suggest that AUAE inhibited the tonic inhibitory signals in the enteric nervous system (e.g., nitric oxide). AUAE and CMAE both enhanced spontaneous intestinal contractions. However, none of the pharmacological tests could provide any indication of whether AUAE and CMAE act on the smooth muscle or enteric neurons. Additional studies would be needed to delineate the mechanisms of action of AUAE and CMAE.

Furthermore, plant extracts did not reduce the contraction induced by CaCl_2_ (25 mM) or KCl (10 mM). In fact, failure to reduce or enhance the effects of CaCl_2_ or KCl by AUAE did not assume that AUAE acts on enteric neurons or smooth muscles. High concentrations of CaCl_2_ and KCl can cause excitation of all excitable cells, including enteric neurons and intestinal smooth muscle cells.

The inability of AUAE to reduce contraction induced by CaCl_2_ and KCl does not necessarily rule out its potential stimulatory effects on smooth muscle. Several factors could contribute to this phenomenon. For instance, the contraction induced by CaCl_2_ and KCl might involve the same signaling pathways that are activated by AUAE. Additionally, the use of CaCl_2_, KCl, serotonin, or histamine as tool agents may not effectively reflect the direct action of AUAE on smooth muscle. To gain more insight into its effects, experiments using a neuronal blocker (e.g., tetrodotoxin), cholinergic receptor antagonists (such as atropine or hexamethonium), histamine receptor antagonists, or serotonin receptor antagonists could provide more specific information. Another possibility is to assess the effects of AUAE on isolated smooth muscle strips in the absence of the myenteric plexus. Based on our findings, it seems that the two extracts do not directly affect either the muscle or the enteric nervous system, suggesting that they might act on other targets that influence intestinal motility.

In fact, KCl causes depolarisation of the membrane and activates voltage‐dependent calcium channels, which leads to an increase in intracellular calcium and the contraction force of intestinal smooth muscle [55]. Any substance that induces contractions is considered to be an activator of voltage‐dependent calcium channels [56]. CaCl_2_, like KCl, induces a depolarisation of the plasma membrane [57] and activates L‐type calcium channels to induce muscle contraction. However, our extracts have no musculotropic or neurotropic effect.

We have also shown in this work that AUAE and CMAE significantly reduced the glucose concentration in rats as compared to control values. Indeed, chlorogenic acids have been shown to influence postprandial blood sugar concentration, glucose tolerance, and glucose absorption from the intestine. They have been found to reduce the intestinal absorption of glucose in rats by encouraging dispersal of the Na+ electrochemical gradient, which draws glucose into the enterocytes [58], and to inhibit the activity of hepatic glucose‐6‐phosphatase, which is implicated in glucose homeostasis [59, 60]. Lacombe et al. could further demonstrate that the phenolic acid tannic acid was able to inhibit Na+/K+‐ATPase, responsible for maintaining the sodium gradient necessary for sodium‐driven glucose transport into enterocytes. In studies by Manzano and Williamson [61] simple phenolic acids such as p‐coumaric acid were shown to decrease the uptake of glucose into Caco‐2 cells. Other flavonoids such as quercetin monoglucosides, luteolin, as well as naringenin significantly inhibited SGLT1‐mediated glucose uptake in vitro.

## 5 Conclusion

In conclusion, the present results suggested that the GI dynamic mechanism of AUAE and CMAE might be the main laxative activity. Moreover, in ex vivo, AUAE and CMAE possess smooth muscle contraction action on isolated mice jejunum and reduce the elevated blood glucose levels, which provide a scientific basis for the clinical use of combined extracts in GI disorders. The identification of the main involved bioactive compounds and their metabolites in rats should be performed in further study.

## Author Contributions

Conceptualization: S.W., K.R., H.S., and B.E; methodology and data curation: S.W., K.R., H.S., and B.E; writing‐original draft preparation: S.W., K.R., and H.S., writing‐review and editing: S.W., K.R., H.S., B.G., and B.E., and supervision and validation: K.R., H.S., and B.E. All authors have read and agreed to the published version of the manuscript.

## Disclosure

Institutional Review Board Statement: The study was conducted according to the guidelines of the Declaration of Helsinki and approved by the Institutional Review Board at the University of Jendouba, Tunisia.

## Ethics Statement

All procedures on animals in this study were compiled with the National Institutes of Health recommendations for the use and care of animals.

## Conflicts of Interest

The authors declare no conflicts of interest.

## Data Availability

Data sharing not applicable to this article as no datasets were generated or analysed during the current study.

## References

[1] T. Boeing , P. de Souza , L. M. da Silva , and A. Gasparotto Junior , “Herbal Medicines in the Treatment of Dyspepsia: An Overview,” Planta Medica 88, no. 8 (2021): 664–677, 10.1055/a-1580-7782. 34474492

[2] N. J. Talley and A. C. Ford , “Functional Dyspepsia,” New England Journal of Medicine 373, no. 19 (2015): 1853–1863, 10.1056/nejmra1501505. 26535514

[3] J. J. Marín‐Peñalver , I. Martín‐Timón , C. Sevillano‐Collantes , and F. J. Cañizo‐Gómez , “Update on the Treatment of Type 2 Diabetes Mellitus,” World Journal of Diabetes 7, no. 17 (2016): 354, 10.4239/wjd.v7.i17.354. 27660695 PMC5027002

[4] L. Di Magno , F. Di Pastena , R. Bordone , S. Coni , and G. Canettieri , “The Mechanism of Action of Biguanides: New Answers to a Complex Question,” Cancers 14, no. 13 (2022): 3220, 10.3390/cancers14133220. 35804992 PMC9265089

[5] Y. Sakar , B. Meddah , M. A. Faouzi , Y. Cherrah , A. Bado , and R. Ducroc , “Metformin‐Induced Regulation of the Intestinal D‐Glucose Transporters,” Journal of Physiology and Pharmacology 61, no. 3 (2010): 301–307. 20610860

[6] C. J. Bailey , C. Wilcock , and J. H. Scarpello , “Metformin and the Intestine,” Diabetologia 51, no. 8 (2008): 1552–1553, 10.1007/s00125-008-1053-5. 18528677

[7] L. J. McCreight , C. J. Bailey , and E. R. Pearson , “Metformin and the Gastrointestinal Tract,” Diabetologia 59, no. 3 (2016): 426–435, 10.1007/s00125-015-3844-9. 26780750 PMC4742508

[8] S. Wahabi , K. Rtibi , C. Abidi , H. Tounsi , A. Ouerghui , and H. Sebai , “Prophylactic Protective Action of Aqueous Extract of Green Oak Acorns on Ethanol‐Induced Acute Injury to Rat Gastroduodenal Mucosa,” Journal of Medicinal Food 25, no. 3 (2022): 303–312, 10.1089/jmf.2021.0076. 35076295

[9] M. A. Benabderrahim , Y. Yahia , I. Bettaieb , W. Elfalleh , and K. Nagaz , “Antioxidant Activity and Phenolic Profile of a Collection of Medicinal Plants From Tunisian Arid and Saharan Regions,” Industrial Crops and Products 138 (2019): 111427, 10.1016/j.indcrop.2019.05.076.

[10] R. Ben Mansour , H. Wasli , R. Serairi‐Beji , et al., “In Vivo Gastroprotective Effect and Biological Potentialities of Six Tunisian Medicinal Plants Using Multivariate Data Treatment,” Plant Biosystems ‐ an International Journal Dealing With all Aspects of Plant Biology 156, no. 1 (2020): 152–163, 10.1080/11263504.2020.1845840.

[11] K. Pallauf , J. C. Rivas‐Gonzalo , M. D. del Castillo , M. P. Cano , and S. de Pascual‐Teresa , “Characterization of the Antioxidant Composition of Strawberry Tree ( *Arbutus unedo* L.) Fruits,” Journal of Food Composition and Analysis 21, no. 4 (2008): 273–281, 10.1016/j.jfca.2007.11.006.

[12] B.‐M. Ruiz‐Rodr'ıguez , P. Morales , V. Fern'andez‐Ruiz , et al., “Valorizationofwild Strawberry‐Tree Fruits (*Arbutus Unedo* L.) Through Nutritional Assessment and Natural Production Data,” Food Research International 44, no. 5 (2011): 1244–1253, 10.1016/j.foodres.2010.11.015.

[13] F. A. Ayaz , M. Kucukislamoglu , and M. Reunanen , “Non‐Volatile and Phenolic Acids Composition of Strawberry Tree ( *Arbutus unedo* L. var.Ellipsoidea) Fruits,” Journal of Food Composition and Analysis 13, no. 2 (2000): 171–177, 10.1006/jfca.1999.0868.

[14] L. Barros , A. M. Carvalho , J. S. Morais , and I. C. F. R. Ferreira , “Strawberry‐Tree, Blackthorn and Rose Fruits: Detailed Characterisation in Nutrients and Phytochemicals With Antioxidant Properties,” Food Chemistry 120, no. 1 (2010): 247–254, 10.1016/j.foodchem.2009.10.016.

[15] I. Oliveira , P. Baptista , R. Malheiro , S. Casal , A. Bento , and J. A. Pereira , “Influence of Strawberry Tree (*Arbutus Unedo* L.) Fruit Ripening Stage on Chemical Composition and Antioxidant Activity,” Food Research International 44, no. 5 (2011): 1401–1407, 10.1016/j.foodres.2011.02.009.

[16] P. Morales , I. C. F. R. Ferreira , A. M. Carvalho , et al., “Wild Edible Fruits as a Potential Source of Phytochemicals With Capacity to Inhibit Lipidperoxidation,” European Journal of Lipid Science and Technology 115, no. 2 (2013): 176–185, 10.1002/ejlt.201200162.

[17] R. Vidrih , N. P. Ulrih , E. Zlati'c , J. Hribar , and Z. Prgomet , “The Nutritional and Physico‐Chemical Properties of Ripe (Ziziphus Jujube) Fruits Grown in Istria,” Acta Horticulturae 840 (2009): 525–528, 10.17660/actahortic.2009.840.74.

[18] M. Seker and C. Toplu , “Determination, and Comparison of Chemical Characteristics of *Arbutus unedo* *L*. and Arbutus and Rachnae L. (Family Ericaceae) Fruits,” Journal of Medicinal Food 13, no. 4 (2010): 1013–1018, 10.1089/jmf.2009.0167. 20553155

[19] H. N. Mrabti , F. M. El Abbes , F. M. Mayuk , et al., “ *Arbutus unedo* L., (Ericaceae) Inhibits Intestinal Glucose Absorption and Improves Glucose Tolerance in Rodents,” Journal of Ethnopharmacology 235 (2019): 385–391, 10.1016/j.jep.2019.02.013. 30742883

[20] A. M. Carvalho , Plantas Y Sabidur'ıaPopular Del Parque Natural De Montesinho: Un EstudioEtnobot'anico En Portug (CSIC, 2010).

[21] J. M. Rigelsky and B. V. Sweet , “Hawthorn: Pharmacology and Therapeutic Uses,” American Journal of Health‐System Pharmacy 59, no. 5 (2002): 417–422, 10.1093/ajhp/59.5.417. 11887407

[22] J. Arrieta , D. Siles‐Barrios , J. Garc'ıa‐S'anchez , B. Reyes‐Trejo , and M. E. S'anchez‐Mendoza , “Relaxant Effect of the Extracts of *Crataegus Mexicana* on Guinea Pig Tracheal Smooth Muscle,” Pharmacognosy Journal 2, no. 17 (2010): 40–46, 10.1016/S0975-3575(10)80008-2.

[23] I. Pawlaczyk‐Graja , “Polyphenolic‐Polysaccharide Conjugates From Flowers and Fruits of Single‐Seeded Hawthorn (*Crataegus Monogyna* Jacq.): Chemical Profiles and Mechanisms of Anticoagulant Activity,” International Journal of Biological Macromolecules 116 (2018): 869–879, 10.1016/j.ijbiomac.2018.05.101. 29777813

[24] H. Bardakci , E. Celep , T. G¨ozet , Y. Kan , and H. Kırmızıbekmez , “Phytochemical Characterization and Antioxidant Activities of the Fruit Extracts of Several Crataegus Taxa,” South African Journal of Botany 124 (2019): 5–13, 10.1016/j.sajb.2019.04.012.

[25] H. Jdir , M. Jridi , M. Mabrouk , et al., “The Rocket, *Diplotaxis Simplex*, as a Functional Ingredient: LC‐ESI‐MS Analysis and Its Effect on Antioxidant and Physical Properties of Bread,” Journal of Food and Nutrition Research 5, no. 3 (2017): 197–204, 10.12691/jfnr-5-3-10.

[26] A. NRCotN , Guide for the Care and Use of Laboratory Animals (National Academies Press, 2011). 21595115

[27] K. Rtibi , S. Selmi , M. A. Jabri , et al., “Effects of Aqueous Extracts From *Ceratonia siliqua* L. Pods on Small Intestinal Motility in Rats and Jejunal Permeability in Mice,” RSC Advances 6 (2016): 44345–44353.

[28] B. Eto , M. Boisset , B. Griesmar , and J. F. Desjeux , “Effect of Sorbin on Electrolyte Transport in Rat and Human Intestine,” American Journal of Physiology 276, no. 1 (1999): G107–G114, 10.1152/ajpgi.1999.276.1.G107. 9886985

[29] B. J. Lamont , Y. Li , E. Kwan , T. J. Brown , H. Gaisano , and D. J. Drucker , “Pancreatic GLP‐1 Receptor Activation Is Sufficient for Incretin Control of Glucose Metabolism in Mice,” Journal of Clinical Investigation 122 (2012): 388–402. 22182839 10.1172/JCI42497 PMC3248276

[30] R. H. Glew , F. A. Ayaz , H. S. Huang , L. T. Chuang , D. J. VanderJagt , and M. Strnad , “Evolution of Fatty Acids in Medlar (Mespilus Germanica L.) Mesocarp at Different Stages of Ripening,” Grasas y Aceites 53, no. 3 (2002): 328, 10.3989/gya.

[31] F. Martinelli , A. Perrone , S. Yousefi , et al., “Botanical, Phytochemical, Anti‐Microbial and Pharmaceutical Characteristics of Hawthorn (*Crataegus Monogyna* Jacq.), Rosaceae,” Molecules 26, no. 23 (2021): 7266, 10.3390/molecules26237266. 34885847 PMC8659235

[32] M. Naveed , V. Hejazi , M. Abbas , et al., “Chlorogenic Acid (CGA): A Pharmacological Review and Call for Further Research,” Biomedicine & Pharmacotherapy 97 (2018): 67–74, 10.1016/j.biopha.2017.10.064. 29080460

[33] P. Ljubuncic , I. Portnaya , U. Cogan , H. Azaizeh , and A. Bomzon , “Antioxidant Activity of Crataegus Aronia Aqueous Extract Used in Traditional Arab Medicine in Israel,” Journal of Ethnopharmacology 101, no. 1–3 (2005): 153–161, 10.1016/j.jep.2005.04.024. 15970411

[34] S. Rodrigues , R. C. Calhelha , J. C. M. Barreira , et al., “ *Crataegus monogyna* Buds and Fruits Phenolic Extracts: Growth Inhibitory Activity on Human Tumor Cell Lines and Chemical Characterization by HPLC–Dad–ESI/MS,” Foodservice Research International 49, no. 1 (2012): 516–523, 10.1016/j.foodres.2012.07.046.

[35] W. Hou , S. Hu , Z. Su , et al., “Myricetin Attenuates LPS‐Induced Inflammation in Raw 264.7 Macrophages and Mouse Models,” Future Medicinal Chemistry 10, no. 19 (2018): 2253–2264, 10.4155/fmc-2018-0172. 30095283

[36] M. Jiang , M. Zhu , L. Wang , and S. Yu , “Anti‐Tumor Effects and Associated Molecular Mechanisms of Myricetin,” Biomedicine & Pharmacotherapy 120 (2019): 109506, 10.1016/j.biopha.2019.109506. 31586904

[37] S. Jiang , X. Tang , M. Chen , et al., “Design, Synthesis and Antibacterial Activities Against Xanthomonas Oryzae Pv. Oryzae, Xanthomonas Axonopodispv. Citri and *Ralstonia Solanacearum* of Novel Myricetin Derivatives Containing Sulfonamide Moiety,” Pest Management Science 76, no. 3 (2020): 853–860, 10.1002/ps.5587. 31419003

[38] R. Ren , S. Yin , B. Lai , et al., “Myricetin Antagonizes Semen‐Derived Enhancer of Viral Infection (SEVI) Formation and Influences Its Infection‐Enhancing Activity,” Retrovirology 15, no. 1 (2018): 49, 10.1186/s12977-018-0432-3. 30012153 PMC6048764

[39] L. Wang , H. Wu , F. Yang , and W. Dong , “The Protective Effects of Myricetin Against Cardiovascular Disease,” Journal of Nutritional Science and Vitaminology 65, no. 6 (2019): 470–476, 10.3177/jnsv.65.470. 31902859

[40] S. Ahmed , H. Khan , M. Aschner , M. M. Hasan , and S. T. S. Hassan , “Therapeutic Potential of Naringin in Neurological Disorders,” Food and Chemical Toxicology 132 (2019): 110646, 10.1016/j.fct.2019.110646. 31252025

[41] C. Guo , G. Xue , B. Pan , et al., “Myricetin Ameliorates Ethanol‐Induced Lipid Accumulation in Liver Cells by Reducing Fatty Acid Biosynthesis,” Molecular Nutrition & Food Research 63, no. 14 (2019): e1801393, 10.1002/mnfr.201801393. 31168926

[42] M. K. Mbayo , E. M. Kalonda , R. K. Muya , et al., “Test D'activité Antimitotique et Étude Chimique Préliminaire de Quelques Euphorbiaceae du Katanga Méridional (RDC),” Phytothérapie 16 (2016): 1–13, 10.1007/s10298-016-1060-5.

[43] T. K. Ha , I. Jung , M. E. Kim , S. K. Bae , and J. S. Lee , “Anti‐Cancer Activity of Myricetin Against Human Papillary Thyroid Cancer Cells Involves Mitochondrial Dysfunction‐Mediated Apoptosis,” Biomedicine & Pharmacotherapy 91 (2017): 378–384, 10.1016/j.biopha.2017.04.100. 28463801

[44] P. Ducrotté , “Phytothérapie et Dyspepsie Fonctionnelle,” Hegel 1, no. 1 (2015): 40–41, 10.4267/2042/56339.

[45] M.‐A. Jabri , D. Wannes , N. Hajji , M. Sakly , L. Marzouki , and H. Sebai , “Role of Laxative and Antioxidant Properties of *Malva sylvestris* Leaves in Constipation Treatment,” Biomedicine & Pharmacotherapy 89 (2017): 29–35, 10.1016/j.biopha.2017.02.020. 28214685

[46] Y. Dey , S. Mahor , D. Kumar , M. Wanjari , S. Gaidhani , and A. Jadhav , “Gastrokinetic Activity of *Amorphophallus Paeoniifolius* Tuber in Rats,” Journal of Intercultural Ethnopharmacology 5, no. 1 (2016): 36–42, 10.5455/jice.20151211063819. 27069720 PMC4805145

[47] H. Doi , R. Sakakibara , M. Sato , et al., “Dietary Herb Extract Rikkunshi‐To Ameliorates Gastroparesis in Parkinson's Disease: A Pilot Study,” European Neurology 71, no. 3‐4 (2014): 193–195, 10.1159/000355608. 24457529

[48] Y. Falkén , D.‐L. Webb , M. Abraham‐Nordling , U. Kressner , P. M. Hellström , and E. Näslund , “Intravenous Ghrelin Accelerates Postoperative GE and Time to First Bowel Movement in Humans,” Neurogastroenterology and Motility 25, no. 6 (2013): 474‐e364, 10.1111/nmo.12098. 23527561

[49] C. Abidi , K. Rtibi , S. Boutahiri , et al., “Dose‐Dependent Action of *Zingiber Officinale* on Colonic Dysmotility and Ex Vivo Spontaneous Intestinal Contraction Modulation,” Dose‐Response 20, no. 3 (2022): 15593258221127556, 10.1177/15593258221127556. 36132707 PMC9483983

[50] A. O. Badary , S. A. Awad , A. M. Sherief , and M. A. Hamada , “In Vitro and In Vivo Effects of Ferulic Acid on Gastrointestinal Motility: Inhibition of Cisplatin‐Induced Delay in Gastric Emptying in Rats,” World Journal of Gastroenterology 12, no. 33 (2006): 5363–5367. 16981269 10.3748/wjg.v12.i33.5363 PMC4088206

[51] J.‐G. Jiang , Q. Luo , S.‐S. Li , et al., “Cinnamic Acid Regulates the Intestinal Microbiome and Short‐Chain Fatty Acids to Treat Slow Transit Constipation,” World Journal of Gastrointestinal Pharmacology and Therapeutics 14, no. 2 (2023): 4–21. 36911598 10.4292/wjgpt.v14.i2.4 PMC9993904

[52] F. Alan , H. Dayioglu , and A. Yilmaz , “Investigation of the Effection Mechanism of Cinnamic Acid on Contraction and Relaxation of Smooth Muscles of Ileum and Bladder of Rats,” Journal of Scientific Reports‐A 44 (2020): 1–23.

[53] M. Cui , Y. Li , T. Zheng , et al., “Efficacy and Molecular Mechanism of Quercetin on Constipation Induced by Berberine via Regulating Gut Microbiota,” International Journal of Molecular Sciences 25, no. 11 (2024): 6228, 10.3390/ijms25116228. 38892414 PMC11173111

[54] M. Tonini , S. Lecchini , G. Frigo , and A. Crema , “Action of Tetrodotoxin on Spontaneous Electrical Activity of Some Smooth Muscle Preparations,” European Journal of Pharmacology 29, no. 2 (1974): 236–240. 4442442 10.1016/0014-2999(74)90021-1

[55] C. Buharalioğlu and F. Akar , “The Reactivity of Serotonin, Acetylcholine, and Kcl‐Induced Contractions to Relaxant Agents in the Rat Gastric Fundus,” Pharmacological Research 45, no. 4 (2002): 325–331, 10.1006/phrs.2002.0950. 12030797

[56] T. Godfraind and R. C. Miller , “Calcium Entry and Calcium Entry Blockade,” Cell Calcium 5, no. 3 (1984): 280, 10.1016/0143-4160(84)90070-8.

[57] M. E. R. Flynn , Ionic Conductances Involved in the Electrical Activity of the Canine Gastrointestinal Tract (University of Nevada, Reno, 1999).

[58] C. A. Welsch , P. A. Lachance , and B. P. Wasserman , “Dietary Phenolic Compounds: Inhibition of Na+−Dependent D‐Glucose Uptake in Rat Intestinal Brush Border Membrane Vesicles,” Journal of Nutrition 119, no. 11 (1989): 1698–1704, 10.1093/jn/119.11.1698. 2600675

[59] W. J. Arion , W. K. Canfield , F. C. Ramos , et al., “Chlorogenic Acid and Hydroxy Nitrobenzaldehyde: New Inhibitors of Hepatic Glucose 6‐Phosphatase,” Archives of Biochemistry and Biophysics 339, no. 2 (1997): 315–322, 10.1006/abbi.1996.9874. 9056264

[60] H. Hemmerle , H.‐J. Burger , P. Below , et al., “Chlorogenic Acid and Synthetic Chlorogenic Acid Derivatives: Novel Inhibitors of Hepatic Glucose‐6‐Phosphate Translocase,” Journal of Medicinal Chemistry 40, no. 2 (1997): 137–145, 10.1021/jm9607360. 9003513

[61] S. Manzano and G. Williamson , “Polyphenols and Phenolic Acids From Strawberry and Apple Decrease Glucose Uptake and Transport by Human Intestinal Caco‐2 Cells,” Molecular Nutrition & Food Research 54, no. 12 (2010): 1773–1780, 10.1002/mnfr.201000019. 20564476

